# Post genomics era for orchid research

**DOI:** 10.1186/s40529-017-0213-7

**Published:** 2017-12-12

**Authors:** Wen-Chieh Tsai, Anne Dievart, Chia-Chi Hsu, Yu-Yun Hsiao, Shang-Yi Chiou, Hsin Huang, Hong-Hwa Chen

**Affiliations:** 1 Institute of Tropical Plant Sciences, National Cheng Kung University, Tainan, 701 Taiwan; 2 Orchid Research and Development Center, National Cheng Kung University, Tainan, 701 Taiwan; 3 Department of Life Sciences, National Cheng Kung University, Tainan, 701 Taiwan; 4 CIRAD, UMR AGAP, TA A 108/03, Avenue Agropolis, 34398 Montpellier, France; 5 Present Address: School of Life Sciences and Biotechnology, Shanghai Jiao Tong University, 800 Dongchuan Road, Life Sciences Building, Room 3-117, Shanghai, 200240 People’s Republic of China

**Keywords:** Comparative genomics, Genome editing, Genome evolution, GWAS, Orchidaceae, *Phalaenopsis*, Post genomics era, Receptor-like kinase, Secondary metabolomics, Terpene synthase

## Abstract

Among 300,000 species in angiosperms, Orchidaceae containing 30,000 species is one of the largest families. Almost every habitats on earth have orchid plants successfully colonized, and it indicates that orchids are among the plants with significant ecological and evolutionary importance. So far, four orchid genomes have been sequenced, including *Phalaenopsis equestris*, *Dendrobium catenatum*, *Dendrobium officinale*, and *Apostaceae shengen*. Here, we review the current progress and the direction of orchid research in the post genomics era. These include the orchid genome evolution, genome mapping (genome-wide association analysis, genetic map, physical map), comparative genomics (especially receptor-like kinase and terpene synthase), secondary metabolomics, and genome editing.

## Background

Containing about 30,000 species, Orchidaceae plants account for one tenth of all angiosperms containing 300,000 species (Hsiao et al. 2011b). More than 70% of orchids are epiphytic growth with distinct physiological characteristics (Hsiao et al. 2011b). Orchids are highly adapted to unfavorable environment. They have colonized successfully every habitat on earth wherever the sun shines. According to the molecular clock revealed by whole genome sequencing of *Phalaenopsis equestris* (Cai et al. 2015), the emergence of orchids have occurred in the late Cretaceous (76 Mya), and allowed them to cross the border of Jurassic mass extinction (66 Mya). This is consistent to the observation of the date for the most ancient fossil record (76–84 Mya) (Ramirez et al. 2007). It has been speculated that the speciation rates of orchids are exceptionally high (Gill 1989), with the fact that, even now, new species of orchids are still recorded worldwide suggesting that the evolution of orchids has never ceased.

**Fig. 1:**
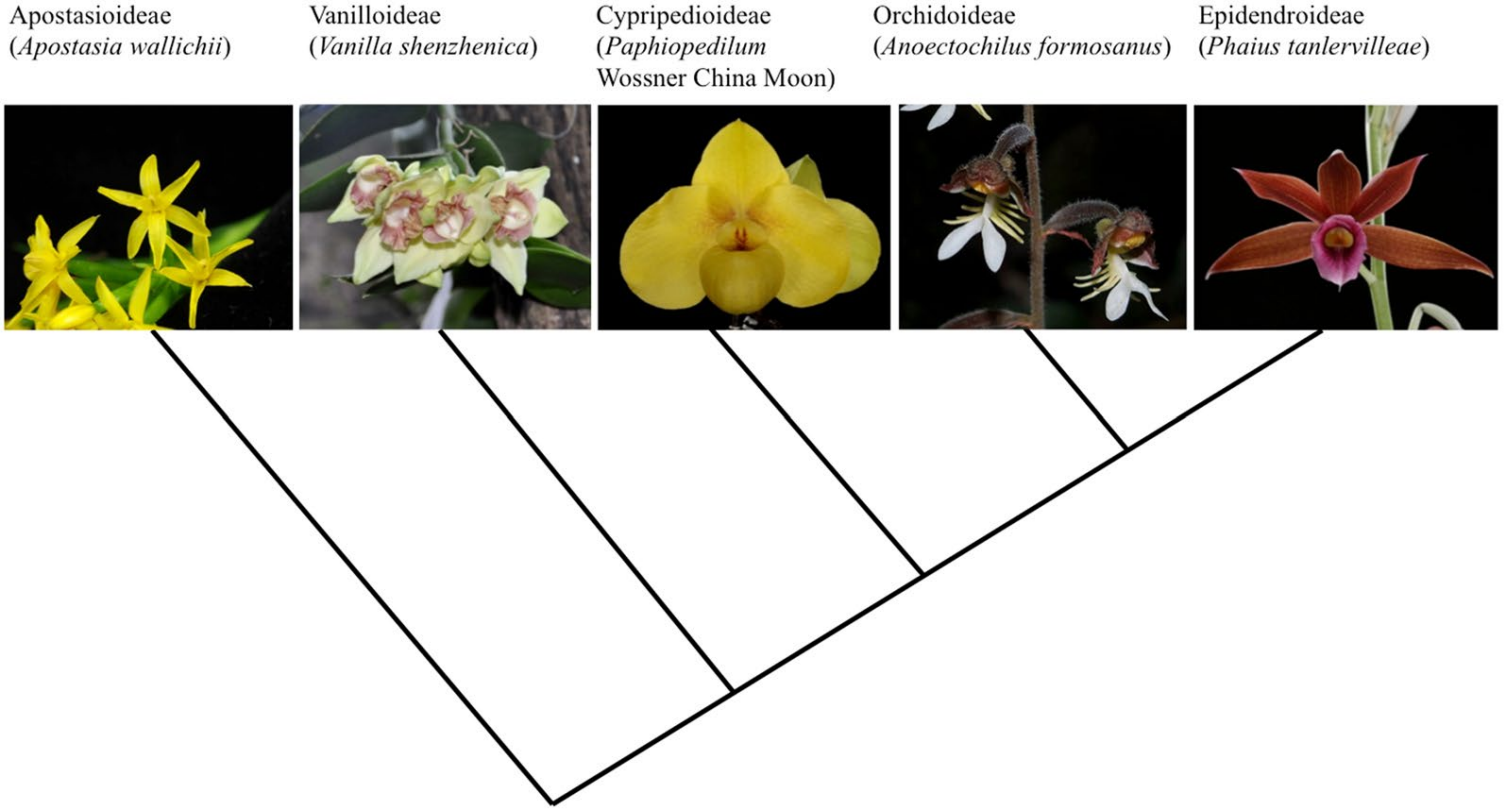
The phylogenetic relationship among five subfamilies of Orchidaceae, and their example plants

The enormous number of Orchidaceae species allows it divided into five subfamilies: Apostasioideae, Vanilloideae, Cypripedioideae, Orchidoideae, and Epidendroideae (Fig. 1). They are all extraordinary floral diversified, and this heterogeneity has been related to the specialized interaction between the pollinators and orchid flowers (Cozzolino and Widmer 2005). The unique features for orchids include the obligate interactions between orchids and mycorrhizal fungi (Otero and Flanagan 2006), with C3 and/or crassulacean acid metabolism photosynthesis (Mascher et al. 2017), and epiphytic growth forms (Silvera et al. 2009). Orchids have exclusive reproductive strategies contributing to their successful adaptation to their ecological exploitations (Yu and Goh 2000).

The orchid cultivation and hybridization in Taiwan is very popular to the worldwide orchid market. The elegant appearance, some even with charming fragrance, and prolonged long life for orchid flowers have promoted attractiveness of *Phalaenopsis* orchids among breeders, nurseries and customers. For the past 20 years, orchid researchers have devoted to establish the foundation of orchid genomics research. These include karyotype analysis (Kao et al. 2001; Lin et al. 2001), genome size analysis (Chen et al. 2013, 2014), establishment of expressed sequence tags (ESTs) (Hsiao et al. 2006), genomics and transcriptomics databases (Fu et al. 2011; Su et al. 2011, 2013; Tsai et al. 2013), bacterial artificial chromosome (BAC) end sequencing (BES) (Hsu et al. 2011), chloroplast genome sequencing (Chang et al. 2006; Pan et al. 2012), miRNA (Lin et al. 2013a), and whole genome sequencing (Cai et al. 2015; Yan et al. 2015; Zhang et al. 2016a, 2017). In addition, orchid functional genomics studies has been available with virus-induced gene silencing using *Cymbidium* mosaic virus infectious clones for the assessing gene functions involved in flower color, floral morphogenesis and floral scent studies (Lu et al. 2007; Hsieh et al. 2013a, b). In micro-propagation, the induction of polyploidy has been developed to circumvent the hybrid incompatibility (Sattler et al. 2016).

In this article, we review the relevant orchid research progress and the future directions of the orchid investigation at the post genomics era. These include the orchid genome evolution, genome mapping, comparative genomics, secondary metabolomics, and genome editing.

## Orchid genome evolution

### Genome size variation

Angiosperms have variable genome sizes ranging nearly 2400-fold from *Genlisea margaretae* (Lentibulariaceae) with just 0.065 pg to the huge genome in *Paris japonica* (Melanthiaceae) with 152.23 pg. Many species with large genomes are observed in monocots, such as species in Alliaceae, Asparagaceae, Liliaceae, Melanthiaceae and Orchidaceae (Leitch et al. 2009). Among these, Orchidaceae with the genome sizes ranging 168-fold (1C = 0.33–55.4 pg) are perhaps the most diverse angiosperm families (Leitch et al. 2009).

As the species-richest subfamily, Epidendroideae with genome contents ranging over 60-fold (1C = 0.3–19.8 pg) harbor the most variable genome size in Orchidaceae. Orchidoideae, where the largest descending/offspringing from species in subtribe Orchidinae, are pictured by a more restricted range of genomes (1C = 2.9–16.4 pg) varying not more than sixfold. Cypripedioideae show genome sizes ranging only tenfold (1C = 4.1–43.1 pg). Cypripedioideae contain the largest mean genome size (1C = 25.8 pg) among all the subfamilies. Only genome size of few species in Vanilloideae was estimated, ranging from 1C = 7.3 to 55.4 pg. In this subfamily, *Pogonia ophioglossoides* presents the largest genome size (1C = 55.4 pg) (Leitch et al. 2009). Apostasioideae, the primitive subfamilies, contain calculated 1C-values ranging from 0.38 pg in *Apostasia nuda* to 5.96 pg in *Neuwiedia zollingeri* var*. javanica*, a close to 16-fold range (Jersáková et al. 2013). *P. equestris* and *P. aphrodite* subsp. *formosana*, the two native *Phalaenopsis* species usually be used as parents for breeding in Taiwan, respectively have a relative small genome size of 1.6 and 1.4 pg/1C (Chen et al. 2013, 2014).

### Recent progress of orchid transcriptomic sequencing

High-throughput EST sequencing provides a gateway into the genome by reasons of the many data covered in the genomewide expression information. Before the next generation sequencing technologies (Zhang et al. 2013) were developed, the most popular sequencing method was the Sanger method, which was applied to the EST sequencing project. 1080 subtractive ESTs were obtained from an *Oncidium* Gower Ramsey pseudobulb subtractive EST library (Tan et al. 2005). Most ESTs were revealed as being related to carbohydrate metabolism and regulatory function, biosynthesis of mannose, pectin and starch, stress-related, and transportation (Tan et al. 2005). To illustrate expressed genes in reproductive organs of *Phalaenopsis*, *P. equestris* mature flower buds were collected and 5593 ESTs were sequenced and annotated (Tsai et al. 2006). In addition, 2359 ESTs were sequenced from scented *P. bellina* flower buds cDNA library to deduced ESTs involved in scent biosynthesis pathway (Hsiao et al. 2006).

The sudden rise of rapid and low-cost next-generation sequencing technologies is substantially promoting our competence to examine the sequences information comprehensively at unparalleled resolution and depth in a cell (Delseny et al. 2010). The technologies were adopted rapidly for orchid transcriptome analysis (Table 1). 454 technology was independently applied to generate 8233 contigs and 34,630 singletons sequenced from the mixed tissues of three *Phalaenopsis* species (Tsai et al. 2013), and 50,908 contig sequences released from six different tissues of *O*. Gower Ramsey (Chang et al. 2011). These data set expansively increased information of expressed genes in *Phalaenopsis* and *Oncidium* and speed identifying sets of ESTs associated with a broad range of biological processes (Chang et al. 2011; Hsiao et al. 2011a; Huang et al. 2015). A total of 121,917 unique ESTs were obtained from the *Ophrys* species by using 454 pyrosequencing and Illumina (Solexa) technologies to identify genes responding to pollinator attraction (Sedeek et al. 2013). A traditional Chinese herb together with 454 pyrosequencing and Illumina technology were able to generate plentiful ESTs for mining the genes participated in alkaloid biosynthetic pathway and polysaccharide biosynthesis in *Dendrobium officinale* (Guo et al. 2013; Zhang et al. 2016b). To provide a general resource for studying the pod development of *Vanilla planifolia*, one of the most valued flavour species for its flavour qualities, the combined 454 and Illumina RNA-seq technologies produced de novo transcriptome with high quality assembly for this important orchid cash crop (Rao et al. 2014). In addition, to improve the horticultural value of *Phalaenopsis* and *Cymbidium*, transcriptome derived from browning leaf of *Phalaenopsis* explant (sequencing by Illumina HiSeq 2000), and variable colour of *Cymbidium* leaf (sequencing by 454 pyrosequencing) were investigated (Xu et al. 2015; Zhu et al. 2015). To study the symbiotic orchid–fungus relationship and the molecular mechanism of orchid seed germination, 454 and Illumina were adopted to explore transcriptomes derived from *Serapias vomeracea* (Perotto et al. 2014), *C. hybridum* (Zhao et al. 2014), *Anoectochilus roxburghii* (Liu et al. 2015), and *Gastrodia elata* (Tsai et al. 2016).

**Table 1:** Characteristics of findings in the literature for the application of next generation sequencing (NGS) to orchid transcriptomes

Subfamily	Species	Sequencing platform	Tissue	Study aim	References
Apostasioideae	*Apostasia shenzhenica*	Illumina/Solexa	Mature flower buds	Study of floral development and evolutionary trends of orchid flowers	Tsai et al. (2013)
Apostasioideae	*Neuwiedia malipoensis*	Illumina/Solexa	Mature flower buds		
Vanilloideae	*Vanilla shenzhenica*	Illumina/Solexa	Mature flower buds		
Vanilloideae	*Galeola faberi*	Illumina/Solexa	Mature flower buds		
Cypripedioideae	*Paphiopedilum armeniacum*	Illumina/Solexa	Mature flower buds		
Cypripedioideae	*Cypripedium singchii*	Illumina/Solexa	Mature flower buds		
Orchidoideae	*Habenaria delavayi*	Illumina/Solexa	Mature flower buds		
Orchidoideae	*Hemipilia forrestii*	Illumina/Solexa	Mature flower buds		
Epidendroideae	*Phalaenopsis equestris*	Illumina/Solexa	Mature flower buds		
Epidendroideae	*Cymbidium sinense*	Illumina/Solexa	Mature flower buds		
Vanilloideae	*Vanilla planifolia*	Illumina/Solexa Roche/454	Pod tissues, seeds	Study of biosynthetic routes to flavor components	Rao et al. (2014)
Cypripedioideae	*Paphiopedilum concolor*	Illumina Hiseq 2000	Roots	Identify the genes that control root growth and development	Li et al. (2015b)
Orchidoideae	*Ophrys* species	Roche/454 Illumina/Solexa	Flowers, labellums, leaves, flower organ from open flowers and buds	Identify genes responding for pollinator attraction	Sedeek et al. (2013)
Orchidoideae	*Orchis italica*	Illumina/Solexa (MiSeq)	Inflorescences	The roles of small RNAs on the flower development	Aceto et al. (2014)
Orchidoideae	*Orchis italica*	Illumina Hiseq 2500	Florets of inflorescence before anthesis	Analyzing transcripts potentially involved in flower development	De Paolo et al. (2014)
Orchidoideae	*Serapias vomeracea*	Roche/454	Protocorms	Investigate the molecular bases of the orchid response to mycorrhizal invasion	Perotto et al. (2014)
Orchidoideae	*Anoectochilus roxburghii* (Wall.) Lindl.	Illumina HiSeq 4000	Dry seeds, seeds from asymbiotic or symbiotic germination	Study of seed germination process	Liu et al. (2015)
Orchidoideae	*Gastrodia elata* Blume	Illumina Hiseq 2000	Vegetative tissues, corms, juvenile tubers	Address the gene regulation mechanism in gastrodin biosynthesis	Tsai et al. (2016)
Epidendroideae	*Phalaenopsis aphrodite*	Sanger: EST	Protocorms	Gene discovery and genomic annotation	Fu et al. (2011) , Hsiao et al. (2011a)
Epidendroideae	*Phalaenopsis equestris*	Sanger: EST	Mature flower buds		
Epidendroideae	*Phalaenopsis bellina*	Sanger: EST	Mature flower buds without column		
Epidendroideae	*Phalaenopsis aphrodite* *Phalaenopsis equestris* *Phalaenopsis bellina*	Roche/454	Mixed tissues		
Epidendroideae	*Phalaenopsis equestris*	Illumina/Solexa	Leaves		
Epidendroideae	*Phalaenopsis aphrodite*	Roche/454 Illumina/Solexa	Leaves, stems, roots, young inflorescences, stalks, flower buds, flowers, germinating seeds	Investigate expressed genes involved in many biological processes of orchids	Su et al. (2011)
Epidendroideae	*Phalaenopsis aphrodite*	Illumina/Solexa	Leaves, stalks, flower buds	Study the roles of small RNAs on the regulation of flowering	An et al. (2011), An and Chan (2012)
Epidendroideae	*Phalaenopsis aphrodite*	Illumina/Solexa	Leaves, roots, flowers, germinating seeds, young inflorescences	Identify species- and tissue-specific miRNAs	Chao et al. (2014)
Epidendroideae	*Phalaenopsis* Brother Spring Dancer ‘KHM190’	Illumina Hiseq 2000	Petals, sepals or labellums from flower buds of wild-type and peloric petal mutant plants	Study regulation of floral- organ development	Huang et al. (2015)
Epidendroideae	*Phalaenopsis sp.*	Illumina Hiseq 2000	Explants	Examine *Phalaenopsis* leaf explant browning	Xu et al. (2015)
Epidendroideae	*Oncidium* ‘Gower Ramsey’	Roche/454	Leaves, pseudobulbs, young inflorescences, inflorescences, flower buds, mature flowers	Identify genes associated with flowering time	Chang et al. (2011)
Epidendroideae	*Oncidium* ‘Gower Ramsey’	Illumina/Solexa	Roots with or without fungus	Study the roles of small RNAs on the interaction between root and the fungus	Ye et al. (2014)
Epidendroideae	*Erycina pusilla*	Illumina/Solexa	Roots, leaves, peduncles, flowers, capsules	Investigate photoperiod-dependent flowering genes	Chou et al. (2013)
Epidendroideae	*Erycina pusilla*	Illumina/Solexa	Roots, leaves, peduncles, flowers, capsules	Study the roles of small RNAs on the regulation of flowering	Lin et al. (2013a)
Epidendroideae	*Cymbidium ensifolium* ‘Tiegusu’	Illumina HiSeq 2000	Flower buds, mature flower	Identify genes associated with floral development	Li et al. (2014)
Epidendroideae	*Cymbidium sinense* ‘Qi Jian Bai Mo’	Illumina HiSeq 2000	Plants in vegetative phase/floral differentiation phase/reproductive phase	Identify genes associated with floral development	Zhang et al. (2013)
Epidendroideae	*Cymbidium hybridum* ‘Golden Boy’	Illumina Hiseq 2000	Roots with or without fungus	Study of orchid-mycorrhizal fungi interactions	Zhao et al. (2014)
Epidendroideae	*Cymbidium ensifolium* ‘Tiegusu’	Illumina/Solexa	Flower bud	Identify miRNAs related to floral development	Li et al. (2015c)
Epidendroideae	*Cymbidium ensifolium* ‘tianesu’	Roche/454	Sepals, petals, labellums, gynostemia from flower buds and mature flowers	Reveal genes associated with floral organ differentiation	Yang and Zhu (2015)
Epidendroideae	*Cymbidium sinense* ‘Dharma’	Roche/454	Roots, leaves, pseudobulbs, flowers	Analyze molecular mechanism underlying leaf-color variations	Zhu et al. (2015)
Epidendroideae	*Cymbidium sinense* *Cymbidium atropurpureum* *Cymbidium mannii*	Illumina Hiseq 2000	Leaves	Explore the evolution and molecular regulation of CAM plants	Zhang et al. (2016c)
Epidendroideae	*Dendrobium officinale*	Roche/454	Stems	Study of alkaloid biosynthesis	Guo et al. (2013)
Epidendroideae	*Dendrobium officinale*	Illumina Hiseq 2000	Juvenile and adult plants	Identify genes associated with polysaccharide synthesis	Zhang et al. (2016b)
Epidendroideae	*Dendrobium officinale*	Illumina Hiseq 2500	Flower, roots, leaves, stems	Study of the regulatory networks of the production and accumulation of the medicinal constituents	Meng et al. (2016)

In Orchidaceae, about 40% species adopt crassulacean acid metabolism (Mascher et al.) to fix carbon dioxide suggesting Orchidaceae to be the largest CAM clade in plants (Silvera et al. 2009). To illuminate the origin and evolution of CAM pathway, transcriptomes derived from leaves of CAM orchids *P. equestris*, *D. terminale* and *C. mannii* were sampled at different time interval and sequenced by Illumine HiSeq 2000 (Deng et al. 2016; Zhang et al. 2016c). Their results showed that key carbon fixation pathway genes might primarily evolve by changes at the transcription level in CAM plants. Several techniques were applied to study the development of spectacular orchid flower morphology. This includes the developing floral transcriptomes originating from *Phalaenopsis* (Hsiao et al. 2011a), *Cymbidium* (Zhang et al. 2013; Li et al. 2014; Yang and Zhu 2015), and *Orchis* (De Paolo et al. 2014). The molecular model of MADS-box genes associated with floral development was proposed and discussed.

Recently, root transcriptome from *Paphiopedilum concolor* was also produced to explore genes involved in orchid root development (Li et al. 2015b). Over 1195 unique genes participating in secondary metabolic pathways, and 609 ESTs involved in plant hormone biosynthesis, and plant signal transduction were revealed. The accumulated transcribed sequences could be directly used to develop microarray platform. It is also a resource for phylogenetic analysis. For example, an developed oligomicroarray harboring 14,732 unique expressed sequences based on the information of ESTs collected from *Phalaenopsis* orchids was applied to compare transcriptomes among sepal, petal, and labellum (Hsiao et al. 2013). Three hundred fifteen single-copy orthologs characterized in the transcriptomes of 10 species distributed in all five subfamilies of Orchidaceae were used to investigate the phylogenetic association of orchids (Deng et al. 2015). The results indicated this strategy appeared to be more reliable and efficient than using a few markers of genes for phylogenic analyses, particularly for those orchids whose DNA sequences are difficult to be amplified or the holomycotrophic species (Deng et al. 2015).

NGS technologies are not only applied to characterize orchid transcriptomes but also used to systematically analyze small RNAs in orchids (Table 1). The roles of small RNAs were studied on the regulation of flowering in *P. aphrodite* and *Erycina pusilla* (An et al. 2011; An and Chan 2012; Chou et al. 2013; Lin et al. 2013a), flower development in *Orchis italica* and *Cymbidium ensifolium* (Aceto et al. 2014; De Paolo et al. 2014; Li et al. 2015c), and interaction between the fungus *Piriformospora indica* with an *Oncidium* hybrid orchid (Ye et al. 2014). Later, comprehensive collection of small RNAs derived from *P. aphrodite* (Chao et al. 2014), and *D. officinale* (Meng et al. 2016) were performed. These efforts provided valuable messages about the expression, composition and function of small RNAs which help us to better understand functional genomics of orchids.

For the storage and manage the massive expressed gene sequences from orchid, OrchidBase was developed to collect the transcriptomic sequences from 11 various tissues/organs of *Phalaenopsis* spp. and flower tissues of 10 species distributed in five subfamilies of Orchidaceae (Fu et al. 2011; Tsai et al. 2013; Niu et al. 2016). Both deep sequencing with ABI 3730, Roche 454 and Illumina/Solexa were applied to generate EST sequences collected in OrchidBase. OrchidBase is generously accessible at http://orchidbase.itps.ncku.edu.tw/. The database delivers a prominent feature of genetic resource for both data mining and experimental researches of orchid biology and biotechnology. Orchidstra (http://orchidstra.abrc.sinica.edu.tw), another orchid transcriptomic database, was developed to collect 233,924 unique contigs of *P. aphrodite* transcriptomic sequences by use of a Illumina/Solexa and Roche 454 platform. Profiling analysis with RNA-Seq was applied to categorize the genes with tissue-tropism expression patterns (Su et al. 2011, 2013). In addition, 50,908 contigs of sequences generated by using Roche 454 platform from various organs of *Oncidium* were assembled into the OncidiumOrchidGenomeBase (http://predictor.nchu.edu.tw/oogb/) (Chang et al. 2011). These EST dataset are valuable for the identification of specificity of orchids, annotation of genes for genomic sequencing, and assistance in the organization of the orchid genome.

### Current status of orchid genome sequencing

With the quick development and lower cost of NGS, whole genome sequencing of non-model species can be implemented. The first milestone was the sequencing of the *Phalaenopsis equestris*, a tropical epiphytic orchid and recurrently be used as parent species for orchid breeding (Cai et al. 2015). The *P. equestris* genome was whole-genome shotgun and sequenced by Illumina technology. The genome size was estimated to 1.16 Gb containing 29,431 predicted protein-coding genes (Cai et al. 2015). Analysis of the *P. equestris* genome showed that the majority of the genome (about 62%) was occupied by repetitive DNAs, mostly transposable elements (TEs). In addition, before the radiation of most of the orchid clades, an orchid-specific paleopolyploidy event was discovered. This species is also the first CAM plant that has been whole-genome sequenced, and the gene family (α carbonic anhydrase) involved in CAM pathway has an obvious expansion. The result suggested that the evolution of CAM photosynthesis in *P. equestris* might associate with gene duplication. In addition, genes located at the heterozygous regions might relate to self-incompatibility. Genes in type II MADS-box clades, including the E-class, C/D-class, B-class AP3 and AGL6 clades, contain extra orthologs than other plant species. These expanded clades are involved in orchid floral organ development that can provide the unique evolutionary paths of these floral organ identity genes accorded with the innovative development of lip and column in orchids. Furthermore, the *Phalaenopsis* genome sequence was practical to identify MYB genes controlling floral pigmentation patterning (Hsu et al. 2015) and TCP genes participated in the ovule development (Lin et al. 2016).

*Dendrobium officinale*, with both ornamental value and therapeutic effects, is the second sequenced orchid plant by joining both the NGS Illumina Hiseq 2000 and the third-generation PacBio machineries (Yan et al. 2015). The assembled genome of *D. officinale* had a predicted gene number of 35,567, and that was higher than that in *Phalaenopis*. For example, the number of B-class MADS-box genes presented in *D. officinale* was much higher than that in *Phalaenopsis* with its four members in B-class AP3-like subfamily, and one member in B-class PI-like subfamily. In contrast, 19 AP3-like genes and five PI-like genes were present in this *Dendrobium* genome. It is possible that the *D. officinale* plants used for the whole genome sequencing were hybrids rather than a native species. Later, another *Dendrobium* species (*Dendrobium catenatum*) was whole genome sequenced by Illumina HiSeq 2000 platform (Zhang et al. 2016a). The predicted 28,910 protein-coding genes were comparable with those of *Phalaenopsis*, and a whole genome duplication event could be shared with *Phalaenopsis*. Adaptation to a wide range of ecological niches of *Dendrobium* might relate to the expansion of many resistance-related genes. In addition, it seems that extensive duplication of genes encoding glucomannan synthase associated with the synthesis of medicinal polysaccharides. It also found that MADS-box gene clades ANR1, StMADS11, and MIKC* were expanded in the *Dendrobium* suggesting the function of these clades might relate to the astonishing diversity of plant architecture in *Dendrobium* (Zhang et al. 2016a).

Most recently, the primitive orchid *A. shenzhenica*, a representative of one of two genera that form a sister lineage to the rest of the Orchidaceae, was whole genome sequenced to provide a reference for improving our understanding of orchid origins and evolution (Zhang et al. 2017). The *A. shenzhenica* genome was sequenced by use of a combination of different approaches including Illumina, PacBio and 10× genomics technologies. The total length of the genome assembly was 349 Mb containing 21,841 protein-coding genes. *A. shenzhenica* shows changes within MADS-box gene classes, which control a diverse suite of developmental processes, during orchid evolution. This study provides new insights on the genetic evidence underlying key orchid innovations, including the development of the labellum and gynostemium, pollinia, and seeds without endosperm, as well as the evolution of epiphytism (Zhang et al. 2017).

### Whole-genome duplication

A striking feature of plant genomes is the whole genome duplication (WGD) has occurred for many times (Cui et al. 2006; Van de Peer et al. 2009). WGD is a major evolutionary force in angiosperm genomes. The complete genome sequences of angiosperm trees have provided information on polyploidy and genome evolution. It is now generally recognized that in the predecessor of all seed plants, one WGD occurred, and in the ancestor of all angiosperms, an extra one happened (Jiao et al. 2011). Furthermore, a hexaploidy event preceded the radiation of core eudicots (Jiao et al. 2012). Whereas a WGD shared by most of the monocots was also suggested (Paterson et al. 2004). Based on the *Phalaenopsis* and *Dendrobium* genome sequences, two turns of WGDs in the *D. catenatum* lineage was inferred from the examination of both the allocation of synonymous substitutions per synonymous site (Ks) throughout all paralogous genes and for duplicated genes sitting in synteny blocks (Zhang et al. 2016a). This most recent WGD event is shared with *Dendrobium*, *Phalaenopsis*, and *Apostasia* and might occur near the Cretaceous–Paleogene (K/Pg) boundary. Putative peaks at older Ks age distribution might point to further ancient WGD events in the monocot lineage that could be shared by monocot ancestors (Cai et al. 2015; Zhang et al. 2016a, 2017).

Since it is suggested that species diversification might be facilitated by WGDs (Van de Peer et al. 2009), it is intriguing to perceive whether the WGD is common with the subfamily Orchidoideae containing 3630 species, and the subfamilies Cypripedioideae, and Vanilloideae, which include 180, and 185 species, respectively. Orchidoideae separated from the Epidendroideae (about 20,000 species including *P. equestris* and *D. catenatum*) about 59 million years ago (Gustafsson et al. 2010). Cypripedioideae and the ancestor of Orchidoideae and Epidendroideae subfamilies are predicted to have disconnected from each other around 68 million years ago (Gustafsson et al. 2010). Fortunately, extensive transcriptomic data sets and/or whole genome sequences from members of these other subfamilies are ongoing. It will be optimistic for uncovering the mystery of orchid genome evolution after more orchid genomic and transcriptomic data obtained.

### Transposable elements

Complements of genomic elements and extraordinary variations in sizes were observed in the genomes of flowering plant containing approximately 300,000 species (Wendel et al. 2016). An obvious route to genome expansion is WGD events, but many species with great genomes are diploid. For example, *Oryza australiensis* and *O. sativa* are from the same genus, however, the former is more than twice the size of the latter due to the addition of ~ 400 Mbp of DNA majorly by three individual retrotransposable element families in the past few million years (Piegu et al. 2006). And thus, the dynamics of transposable element proliferation and clearance might play the important role in the majority of variation in plant genome size, which is in turn superimposed by the history of WGD (Bennetzen and Wang 2014).

The assembled *P. equstris* genome is composed of about 62% repetitive DNA. This is relatively higher than those found in rice (29%) and grape (41%), but similar in sorghum (61%). About 59% of the genome is occupied by interspersed repeats and TEs and 3% is accounted for tandem repeats. Among the TEs, ~ 46% of the genome is occupied by long terminal repeats (LTRs). Long interspersed nuclear elements (LINEs) account for ~ 8% of the genome (Cai et al. 2015). Most LTRs (71%) appeared in the *P. equstris* genome during 11.7–43 million years ago, far behind the origin of the last common ancestor of orchids (74–86 million years ago). *Copia* and *Gypsy* TEs might experience a recent burst (Cai et al. 2015).

About 78.1% of the *D. catenatum* genome is occupied by 789 Mb repetitive elements. Retrotransposable elements constitute a huge portion of the *D. catenatum* genome including LINE/RTE (5.68%), LINE/L1 (8.44%), LTR/Gypsy (18.49%), and LTR/Copia (27.36%). In addition, the quantity of de novo predicted repeats was predominantly more than that composed based on Repbase, demonstrating that *D. catenatum* has arose numerous unique repeats contrasted to other sequenced plant genomes (Zhang et al. 2016a). During the last 5 million years, a burst of LTR activity was observed, suggesting that these LTRs were integrated into the genome of *D. catenatum* after it was separated from *P. equestris* (22.6–59.6 million years ago) (Zhang et al. 2016a).

Angiosperms have a high frequency of hybridization and polyploidy, with resultant increased TE activity and genome shuffling, and profitable gene duplication (Oliver et al. 2013). TE activity enhanced genomic plasticity provides a more satisfactory and complete explanation to “abominable mystery” described by Darwin. Although explanations for orchid diversity are persuasive and valid, the extreme diversity of orchids still cannot fully understand. However, due to a few of genomic data of orchids, the role of TE involved in orchid diversity and evolution has not been accessed yet. Probably, artificial arena of *Phalaenopsis* orchid breeding might supply direct and relatively recent clues for the pivotal of TEs in the generation of selectable cultivars in *Phalaenopsis* orchids.

## Genome mapping

Several fundamental studies are necessary to well-assemble the whole-genome sequences. These studies include high-density genetic map and bacteria artificial chromosomes (BACs) or yeast artificial chromosomes (YACs)-based physical map.

### Genetic map

Even though the whole-genome sequence of *Phalaenopsis equestris* is published (Cai et al. 2015), the current scaffold number of *P. equestris* genome is still high (236,185 scaffolds), and the assembly of *P. equestris* scaffolds remains to be improved. A saturated genetic linkage map is regarded as an effective tool to facilitate scaffolds assembly. Besides, genetic mapping is referred to a step-in genome mapping, which can integrate with a physical map and a cytogenetic map to access genes in genome. For many important crops, genome mapping using genetic linkage maps, physical maps and cytogenetic maps are well developed, such as rice, wheat, barley, etc. (Kurata et al. 1994; Ramsay et al. 2000; Hearnden et al. 2007; Li et al. 2015a). In Orchidaceae, the genetic linkage maps have been constructed for the medicinal orchids, *Dendrobium* (Xue et al. 2010; Lu et al. 2012a, b; Feng et al. 2013), but not for the ornamental orchids, *Phalaenopsis*. Hence, it is vital needed to construct a saturated genetic map for *Phalaenopsis.*

Usually, a genetic linkage map is constructed based on the crossing over events during meiosis. By estimating the recombination frequency between molecular markers, linkage groups can be built while several markers are linked together. A genetic linkage map is comprised of the numbers of linkage groups corresponding to the chromosome numbers.

First, to identify the crossing over events, one of the significant factors is a segregated mapping population. For constructing genetic maps in rice and wheat, F_2_ population or recombinant inbred lines (RILs) are often developed as suitable mapping populations (Kurata et al. 1994; Li et al. 2015a). However, unlike other crops, *Phalaenopsis* orchids have long juvenile periods. It usually takes 2–3 years to generate a new generation. Consequently, it is inefficient and time-consuming to generate *Phalaenosis* F_2_ population or RILs. As an alternative approach, an outcrossing F_1_ population is used as a mapping population. Yet, the segregation pattern may be more complex due to the segregated alleles that may vary in number. Currently, a F_1_ population with 116 progenies from the cross between *P. aphrodite* subsp. *formosana* and *P. equestris*, the two native species in Taiwan and important parents in breeding, has been adopted as the mapping population for constructing a genetic map for *Phalaenopsis*.

After determination of the mapping population, various types of molecular markers are applied for linkage analysis. For instance, restriction fragment length polymorphisms (RFLPs), amplified fragment length polymorphisms (AFLPs), expressed sequence tags (ESTs), and simple sequence repeats (SSRs) are widely utilized in the constructing genetic maps for several crops. Among them, SSR markers particularly have benefits for classification and mapping of genes due to their extraordinary reproducibility, co-dominant inheritance, relative large quantity, extreme polymorphism, simplicity of genotyping and easily amplified through PCR (Varshney et al. 2005). Previously, 950 SSRs from *P. equestris* have been identified, and among them 206 SSRs primer sequences were developed for *Phalaenopsis* genetic mapping (Hsu et al. 2011). *P. equestris* contains 38 chromosomes (2N = 2X = 38) and its genome size is 1600 Mb per haploid genome. It is a relatively large genome compared to other crops, *ex.:* rice 400–430 Mb, and sorghum 750–770 Mb (Eckardt 2000). Hence, development of abundant molecular markers for *Phalaenopsis* genome mapping is necessary. Nowadays, as NGS technologies are rapidly developed and spread, huge amount of binary single nucleotide polymorphism (SNP) markers is easily generated. Due to this characteristic of SNP markers generation, constructing a saturated genetic map in a relative short time is feasible.

After a saturated, high-density *Phalaenopsis* genetic map is plotted, it is capable of sequence alignment and boosting *P. equestris* scaffolds assembly. In addition, a genetic linkage map can be adopted for useful trait analysis and applied for quantitative trait loci (QTL) mapping. Combining the phenotypic data of the mapping population, QTLs may be able to be mapped on the saturated genetic linkage map. Moreover, after QTL mapping, the molecular markers corresponding to QTLs will be adopted for marker-assisted selection (MAS) for interested agronomic traits. For orchid marketing, *Phalaenopsis* breeders usually constant cross or backcross for about 5–10 generations to select potentially commercial cultivars. Considering 2–3 years for each generation, a period up to 20–30 years is required to breed valuable and popular cultivars, such as *P.* Sogo Yukidian “V3” and *P.* I-Hsin Sesame, for markets. By applying the saturated genetic map to MAS, the time-consuming breeding process will be shortened. Thus, a saturated genetic map is considered as the first step for breeding by design and to reveal interested traits controlled by QTLs and genes.

### Physical map

The construction of physical map is using restriction enzymes (RE) digestion of large insert clone libraries, such as BACs and YACs (Chaney et al. 2016). For example, the BAC libraries are treated with one RE, and the digested fragment patterns are analyzed by using electrophoresis. Different BAC clones with similar fragment patterns mean that they contain overlapping regions and can be assembled together to form fingerprinting contigs (FPCs). Therefore, physical map provides a framework for accelerated position cloning of genes for important agronomic traits, and assembly of genome sequence. With the integration of genetic map, the molecular markers identified on the genetic map can be aligned with physical map, and show the physical location of these markers.

Alternatively, optical mapping is another approach to construct a physical map. Similar to FPCs, optical mapping uses RE to digest DNA and stained with an intercalating dye. Then DNA fragments are imaged, separated according to their sizes, and then ordered with restriction map. Although optical mapping is effective, it is too complex and slow in lengthening the DNA, data imaging and processing. Hence, the optical mapping has mainly applied for small genome organisms, ex.: bacteria (Zhou et al. 2004; Haas et al. 2009). Further improvement of automatic technology for imaging and data processing is now available, so that optical mapping is granted for mapping of large genomes, such as human genome (Jo et al. 2009; Teague et al. 2010).

As an improved high throughput layout, the BioNano Genomics Irys system is much efficient than the traditional optical mapping. This system uses a modified RE to introduce DNA breakage on a single strand or incisions in DNA at sequences-specific site. The breaks are then labeled with fluorescence, and the DNA molecular is passed through automated electrophoresis system to form linearized DNA in nanochannels. For each molecular, the length and distances between labeled sites are measured and analyzed to create a molecular map. Two DNA molecules containing similar distance patterns are assembled into a large contig. Therefore, the BioNano Genomics Irys system is automated and more efficient for mapping complex plant genomes, including wheat and barley (Xiao et al. 2015; Stankova et al. 2016; Mascher et al. 2017). The physical mapping of *Phalaenopsis* using the upmost technology is currently underway.

### Genome-wide association studies (GWAS)

Plant breeding is the practice to produce novel varieties with better phenotypes which are then applied in farming and husbandry, and for human utility (Pawełkowicz et al. 2016). The basic strategy for plant breeding is to select the best individuals and their progenies containing the desired agricultural traits. The association mapping strategy commonly evaluates the statistical analyses to calculate the significance of the association between various phenotypes and the genetic polymorphism in a set of defined persons with genetic variations (Ogura and Busch 2015).

In the past, a number of molecular markers have already been applied for genome mapping or association analysis, such as SSRs, AFLPs, and various kinds of SNPs. With the emergence of NGS technologies, genome-wide association studies (GWAS) is developed for the application of genetic variations that are largely and compactly distributed all over the genome. Therefore, GWAS has replaced the traditional mapping strategy with more opportunities to identify large collections of molecular markers and find new genes and regulatory sequences responsible for specific traits (Pawełkowicz et al. 2016).

With the great progresses in the methods of detecting trait differences and genetic variations, GWAS has emerged as a promising approach to inspect the relationships between complex genetic polymorphisms and distinctive phenotypes for most species (Slate et al. 2009). Over last decades, the employment of the automated image system encourages high-throughput phenotyping of a lots of plants by using large-scale image snapping and phenotypic trait quantification from the images (Ogura and Busch 2015). GWAS can combine the quantitative traits to their genetic variations in distinct individuals, and inspire the quantitative regulation of the growth and development.

As NGS technologies emerge, the genotyping-by-sequencing (GBS) approach rapidly gets hundreds of thousands to millions of markers to construct the high-resolution genetic map, and generates sufficient information and coverage of plant genomes (Edwards and Batley 2010). GWAS through the GBS approach provides major assistant for traditional crop improvement with the identification of great amount of SNP molecular markers and the production of genetic linkage maps with high-density markers (Poland and Rife 2012).

GBS method is a useful and powerful approach to rapidly get lots of markers to construct genetic maps with high resolution. This tactics generates sufficient information and coverage to the plant genomes (Edwards and Batley 2010). The first step of GBS is to digest genome DNAs from different plants in a population of genetically distinct individuals with an appropriate RE (Elshire et al. 2011). Each digested genome DNA is ligated with unique, short DNA sequences (barcodes adaptors) to make different plant DNAs be assembled and sequence determined together in a sole sequencing lane. Then the genome DNAs were recovered and prepared for NGS to characterize high amount of SNPs between each individual in this population, and these SNPs are applied as molecular markers for the construction of a genetic map with high density (Elshire et al. 2011).

There are three advantages by using GBS for genome-wide genetic analysis. First, the usage of unique short barcode for each plant sample makes all the DNAs be pooled together for sequencing. Second, an appropriate RE is necessary for getting highly reproducible results and important genome regions, as well as reducing the repetitive regions and genome complexity. Third, with the NGS technology, a high amount of SNP molecular markers will be identified from the sequencing data, and be applied to construct a genetic map with high density.

For example, by using the GBS approach, 41,371 SNP markers were identified in 254 advanced wheat breeding lines from the wheat breeding program of International Wheat and Maize Improvement Center (Poland et al. 2012b). In addition, 12.4 Gb of high-quality genome sequence and 129,156 SNP markers have been identified for potato. These SNPs were mapped to 2.1 Mb of potato reference genome, and the average read depth was 63× coverage per cultivar (Uitdewilligen et al. 2013). In durum wheat, 9983 putative SNP markers were characterized between their two parents and used for genotyping 91 RILs (van Poecke et al. 2013).

Moreover, GBS approach is a powerful method to develop high-density markers even lack of the sequenced reference genome or previous identification of DNA polymorphism. The high-density markers identified from GBS can be used to anchor the orders of physical maps as well as the whole genome shotgun sequence to improve the genome assembly (Poland et al. 2012a). The application of genomics-assisted breeding to valuable cash crops especially with complex genomes is a very important process. Similar transitions are found in rapeseed, lupin (Fabacea), lettuce, switchgrass (perennial warm season bunchgrass in north America), soybean, and maize (Bus et al. 2012; Poland and Rife 2012; Truong et al. 2012; Yang et al. 2012; Lu et al. 2013; Sonah et al. 2015). For example, in *Manihot esculenta* Crantz (ICGMC 2015), the complex 2412-cM map is incorporated into 10 biparental maps (containing 3480 meioses), and 22,403 genetic markers are anchored on 18 chromosomes (ICGMC 2015). This map was arranged 71.9% of the draft genome assembly and 90.7% of the predicted protein-coding genes (ICGMC 2015). It is beneficial with the chromosome-anchored genome sequence for breeding amendment by providing the prominent characterization of markers linked to chief traits.

In *Phalaenopsis* orchids, the GBS analysis has been carried out in the first generation offsprings (F_1_) from the cross between *P. aphrodite* subsp. *formosana* (white-flower) and *P. equestris* (red-flower). This is beneficial to the assembly of whole-genome sequences of *P. equestris* into 19 linkage groups (unpublished data). The regulatory regions for various phenotypes in the F_1_ plants, including plant size, flower color and size, have been investigated (unpublished data).

Most economically important traits are usually inherited in a quantitative manner, such as the height, weight, and stress resistance. The genetic basis of the quantitative traits are regulated by many genes, the synergistic effects of genes, and the mutual interactions between genes and environment. The major challenge to identify the QTL is to precisely locate the gene via high-density linkage map. This is an extremely costly and time-consuming process. GWAS with NGS technologies offer an opportunity for a powerful strategy to identify large numbers of SNP markers, and construct high-density genetic association map that is suitable for underling these important and complex QTLs. For example, complete genotyping of 2815 maize inbred accessions have characterized 681,257 SNP markers distributed across all over the maize genome. Among them, SNPs associated to the plausible candidate genes are identified for kernel color, sweetness, and flowering time (Romay et al. 2013).

The orchid floral scent trait could be regulated by QTL. Recently, GBS has been applied to study the deceptive orchid *Orphis*, and found several SNPs linked to odour related genes (Sedeek et al. 2014). The study of the ecological speciation was surveyed to understand the reproductive hurdles and variations in floral phenotypes in four closely related species, sexually deceptive *Ophrys* species with various flower morphology and distinct labellum coloration. Regardless of the flower odour chemistry may fundamentally cause the reproductive hurdles, GBS showed common polymorphism all over the *Ophrys* genome but highly distinguished polymorphisms in genes involved in floral odour biosynthesis (Sedeek et al. 2014). This finding suggests that these species are marked mostly by genic divergence rather than genome-wide variations. This result may be applied to other orchids with sexual deception, where floral odour genes may be amongst the first to differentiate (Sedeek et al. 2014).

Recently, the molecular omics, such as epigenome, transcriptome, proteome, and metabolome, have been used as complementary manners to improve the SNP-trait association studies. Epigenomic changes, that are responded to environment stimulate and reflect in phenotypic changes, provide another meaning of the “molecular phenotype” definition. Recent advances in population epigenomics studied the association between genetic SNPs and epigenomic variations in a global accession collection of *Arabidopsis thaliana* (Kawakatsu et al. 2016). Moreover, the transcriptomic variations, which reflect the variation in both genetic and epigenetic regulatory and other omics studies of the proteome and metabolome, have proved to be great resources as molecular phenotype (He et al. 2013).

Alternatively, it would also be valuable if the epigenome, transcriptome, proteome, and metabolome are applied as molecular markers, the “genotypes”, to calculate their associations to the downstream phenotypic traits. For example, the differential transcriptomes from 368 maize diverse inbred lines was used to identify the expression presence/absence variation (ePAV; genes were expressed, or not) and served as as “phenotype” to perform association analysis with 15 agronomic phenotypes and 526 metabolic traits (Jin et al. 2016). As for the quantitative traits of QTLs, the genomic variations from these molecular omics are also quantitative and range mutable. However, they are not only binary or with limited numbers of alleles. Therefore, the quantitative GWAS (qGWAS) has recently been proposed to solve the continuous genotype issue. It has also been used to explore the regulatory network by treating expression level of genes as both “genotypes” and “phenotypes” (Wen et al. 2016).

The major goal for plant breeding is to select the best progenies with the desired traits. GWAS can guild the genetic variations with the high-density SNP markers for mapping the regulatory locus of these traits. In *Phalaenopsis* orchids, several agricultural traits have been emphasized, including flower sizes, length of floral inflorescence, flower colors and pigmentation patterning, and floral fragrances. With the GWAS analysis, it will be plausible to construct the high-density genetic map, and the regulatory regions responsive for the important traits can be identified. Moreover, this will be beneficial for the *Phalaenopsis* breeding for new varieties with the traits of interests.

## Comparative genomics

### Receptor-like kinases in orchid genomes

Phosphorylation is a reversible addition of phosphate to proteins. This post-translational modification is involved in all signaling pathways and every cellular activity in living organisms. In eukaryotes, this process is conducted by a superfamily of protein kinases (ePKs for eukaryotic protein kinases) which represent ~ 1.5 to 2.5% of all genes in average, making ePK one of the largest protein families in eukaryotes (Manning et al. 2002). In *Arabidopsis thaliana* and rice (*Oryza sativa*), the two model plant species, ~ 1000 and ~ 1500 Ser/Thr protein kinases have been identified, corresponding to 2.9 and 2.3% of the proteomes in respective organisms (http://plantsp.genomics.purdue.edu/index.html) (Shiu et al. 2004; Dardick et al. 2007).

According to the taxonomy of Hanks and Hunter and the studies based on kinase domain similarity and phylogeny of eukaryotes, the kinomes of many eukaryotic genomes have been divided into subfamilies (Hanks and Hunter 1995; Martin et al. 2009). In the plant kingdom, the largest subfamily is composed of plant-specific receptors named receptor-like kinase (RLK) (Shiu et al. 2004; Dardick et al. 2007). These receptors play important roles in signal transduction to relay external signals across cell membranes. They are involved in developmental processes and/or represent guard molecules which are able to recognize pathogen attacks in a process called pathogen-associated molecular patterns (PAMP)-triggered immunity (PTI) (Boller and Felix 2009; Gish and Clark 2011; Wu and Zhou 2013; Antolín-Llovera et al. 2014; Haruta and Sussman 2017). Many of the RLKs are also activated under a whole range of abiotic stress responses (Ye et al. 2017). These receptors typically contain an amino-terminal extracellular domain (ECD), a transmembrane (TM) domain, and an intracellular domain composed of the kinase domain (KD). Several phylogenetic studies of the RLK subfamily have been conducted. They focus mainly on *Arabidopsis* but also on other plant species (Shiu and Bleecker 2001, 2003; Shiu et al. 2004; Lehti-Shiu et al. 2009; Liu et al. 2009; Sakamoto et al. 2012; Zan et al. 2013). *Arabidopsis* RLK phylogenetic analyses inferred from KD alignments have led to the classification into 12 subgroups. These subgroups contain similar motifs in their ECD, including CRINKLY 4 like (CR4L), CrRLK1L (named after the first member identified in *Catharanthus roseus*), cystein-rich (CRK), extensin, lectin (Pawełkowicz et al.), leucine-rich repeats (LRR), lysin motif (LysM), proline-rich extensin-like (PERK), RKF3, wall-associated (Kawakatsu et al.), LRK10L-2 and receptor-like cytoplasmic kinase (RLCK) (Shiu and Bleecker 2001, 2003; Shiu et al. 2004).

**Fig. 2:**
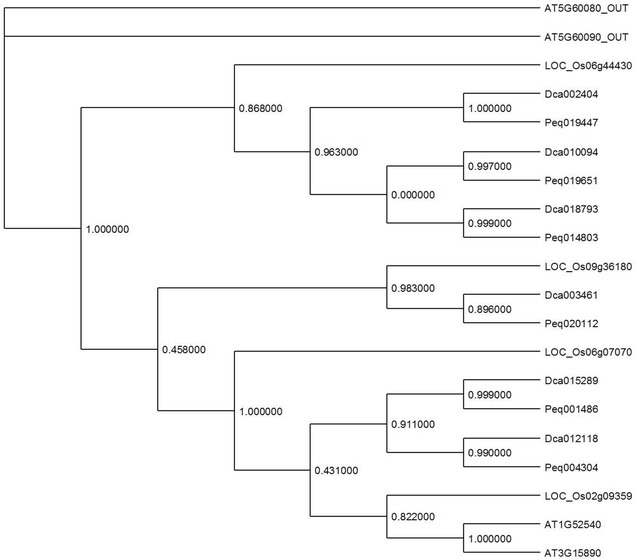
Phylogenetic tree of the RLCK-XV SG. The phylogenetic tree of the RLCK-XV classified sequences has been built with PHYML (default parameters,) (Guindon et al. 2009) based on the MAFFT (Katoh and Standley 2014) alignment of full-length amino-acid sequences. Two Arabidopsis sequences (noted “OUT”) have been added as outgroup to build the tree

To analyze the RLK subfamily in *Dendrobium catenatum* and *Phalaenopsis equestris*, first the hmmsearch program was used to seek the kinase hidden Markov model (HMM) profile (PF00069.16) within the Arabidopsis, rice, and the two orchid proteomes (Sonnhammer et al. 1998; Eddy 2009). The KD sequences of these proteins were extracted and aligned using the MAFFT program (Katoh and Standley 2013). A phylogenetic tree was then built by the maximum likelihood method FastTree (Price et al. 2009). The tree leaves were annotated according to previous Arabidopsis and rice annotations to classify orchid sequences within one of the RLK subgroups (Fig. 2) (Shiu and Bleecker 2003; Shiu et al. 2004). This analysis confirms previous observations that with ~ 650 and ~ 1100 RLK genes in *Arabidopsis* and rice respectively. The expansion rate in rice is approximately twice that of *Arabidopsis*. The subgroup expansions observed in rice have been shown to mainly involve receptors with roles in PTI, like the WAK or LEC subfamilies (Lehti-Shiu et al. 2009; Vaid et al. 2013; Delteil et al. 2016). In the orchid genomes, ~ 400 and ~ 300 RLKs have been detected in *Dendrobium* and *Phalaenopsis* respectively, accounting for 1.4 and 1% of the respective proteomes (containing 29,400 and 29,679 annotated proteins) (see Table 2 for the complete list of accessions and their classification). Compared to the Arabidopsis and rice proportion of RLKs in genomes (~ 1.8 and ~ 1.7% respectively), the number of RLK in orchid genomes is lower. Considering that the *Phalaenopsis* proteome has been established on ~ 85% of the estimated total genome, ~ 15% more RLK sequences could be present in the *Phalaenopsis* genome, increasing the proportion of RLK in this genome to ~ 1.1%. Nevertheless, these proportions are below the range of what has been observed previously in other plant genomes. These results suggest that the *Dendrobium* and *Phalaenopsis* RLK subgroups did not experience large-scale expansions such as the ones observed in the rice genome. Looking at small-scale expansions, it has to be noted that one RLCK clade seems to have expanded specifically in the orchid genomes. Indeed, in the Arabidopsis and rice genomes, 2 and 4 genes are classified into the RLCK-XV subset, while 6 genes belonging to this SG have been retrieved in each orchid genome. The RLCK SG has a particularity among the RLKs since these receptors are lacking the ECD and TM domains. Some of them have been shown to be membrane-anchored and physically associated with membrane-spanning RLK. These protein kinases are then involved in signal relays (not signal perception) via transphosphorylations with other receptors to modulate signaling related to development and stress responses (Lin et al. 2013b). In Arabidopsis, the function of the two genes belonging to the RLCK-XV SG has not been characterized yet. In silico expression data show that these genes are ubiquitously expressed along all developmental stages (Hruz et al. 2008).

**Table 2:** Proportion of *Arabidopsis*, *Oryza* and orchid sequences belonging to RLK subgroups

RLK subgroups	*Arabidopsis thaliana*	*Oryza sativa*	*Dendrobium catenatum*	*Phalaenopsis equestris*
LEC	8.8	19.1	8.9	6.3
CR4L	1.3	1.9	1.7	1.7
CRK	11.6	7.8	8.6	7.3
CrRLK1L	2.4	1.6	3.2	3.3
Extensin	0.5	0.4	1.5	0.3
LRK10L-2	2.1	4.6	2.7	0.7
LysM	1.0	1.0	1.5	2.0
PERK	2.9	1.8	2.2	4.0
RKF3	0.3	0.3	0.5	0.7
LRR	39.3	33.7	38.7	37.2
WAK	4.5	11.9	2.5	2.7
RLCK	24.4	15.4	26.8	32.2

### Terpene synthases (TPSs) in orchid genomes

Terpenoids are the largest class of metabolites found to date, including over 40,000 structures (Croteau et al. 2000; Chappell 2002; Gershenzon and Dudareva 2007), responsible to the interaction between plants and environment, including defense to insects, pathogens, diseases and other stresses, also work to attract pollinators. These compounds are now utilized as pharmaceuticals (e.g., taxol), fragrance (e.g., limonene from orange or lemon oil), industrial materials (e.g., diterpene resin acid), and even as biofuel ingredient (Chen et al. 2011). With the great use of the terpene, understanding the genes that related to the biosynthesis of terpenes is now under study. Terpene synthases (TPSs), the key enzymes that generate structure diversity of terpene are usually followed by cytochromes P450 (CYPs), which further modify the products from TPSs and cause much more diverse molecules of terpenes.

The *TPS* gene family number are different among species, *Physcomitrella patens* (moss) has only one functional *TPS* gene, while in *Eucalyptus grandis* (flooded gum) 113 putative functional genes are found (Külheim et al. 2015). However, through the analysis of plant genome, researchers have shown most of the plant *TPS* gene family have their gene numbers ranging from 20 to 150, belonging to mid-size family (Chen et al. 2011). The functions of TPSs can be mono- or multi-functional, and the enzymes can be highly identical to each other. For instance, the diterpene synthases levopimaradiene/abietadiene synthase and isopimaradiene synthase showed 91% identity in Norway spruce, moreover, the functional bifurcation of these two enzymes was proved to cause by four amino acid residues only (Keeling et al. 2008). This suggests that *TPS* genes have gone through gene duplication, neofunctionalization, and/or subfunctionalization, so that lead to specialized metabolites of large family of *TPS* genes.

**Fig. 3:**
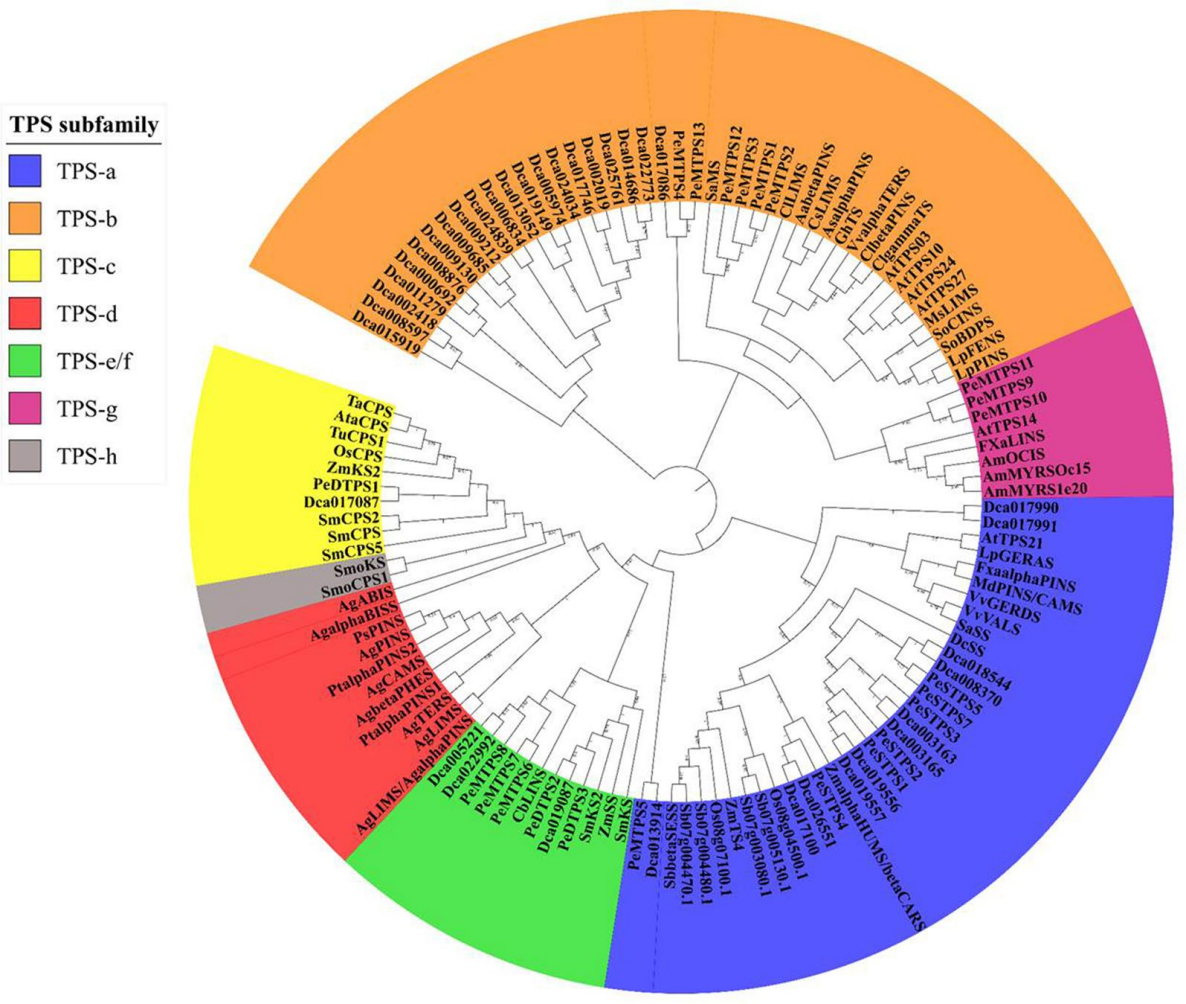
Phylogeny of *P. equestris* and *Dendrobium catenatum’s TPS* subfamily. The evolutionary history was inferred using the Neighbor-Joining method (Saitou and Nei 1987). The bootstrap consensus tree inferred from 100 replicates (Felsenstein 1985) is taken to represent the evolutionary history of the taxa analyzed (Felsenstein 1985). Branches corresponding to partitions reproduced in less than 50% bootstrap replicates are collapsed. The evolutionary distances were computed using the JTT matrix-based method (Jones et al. 1992) and are in the units of the number of amino acid substitutions per site. The analysis involved 123 amino acid sequences. All ambiguous positions were removed for each sequence pair. There were a total of 1411 positions in the final dataset. Evolutionary analyses were conducted in MEGA7 (Kumar et al. 2016)

**Fig. 4:**
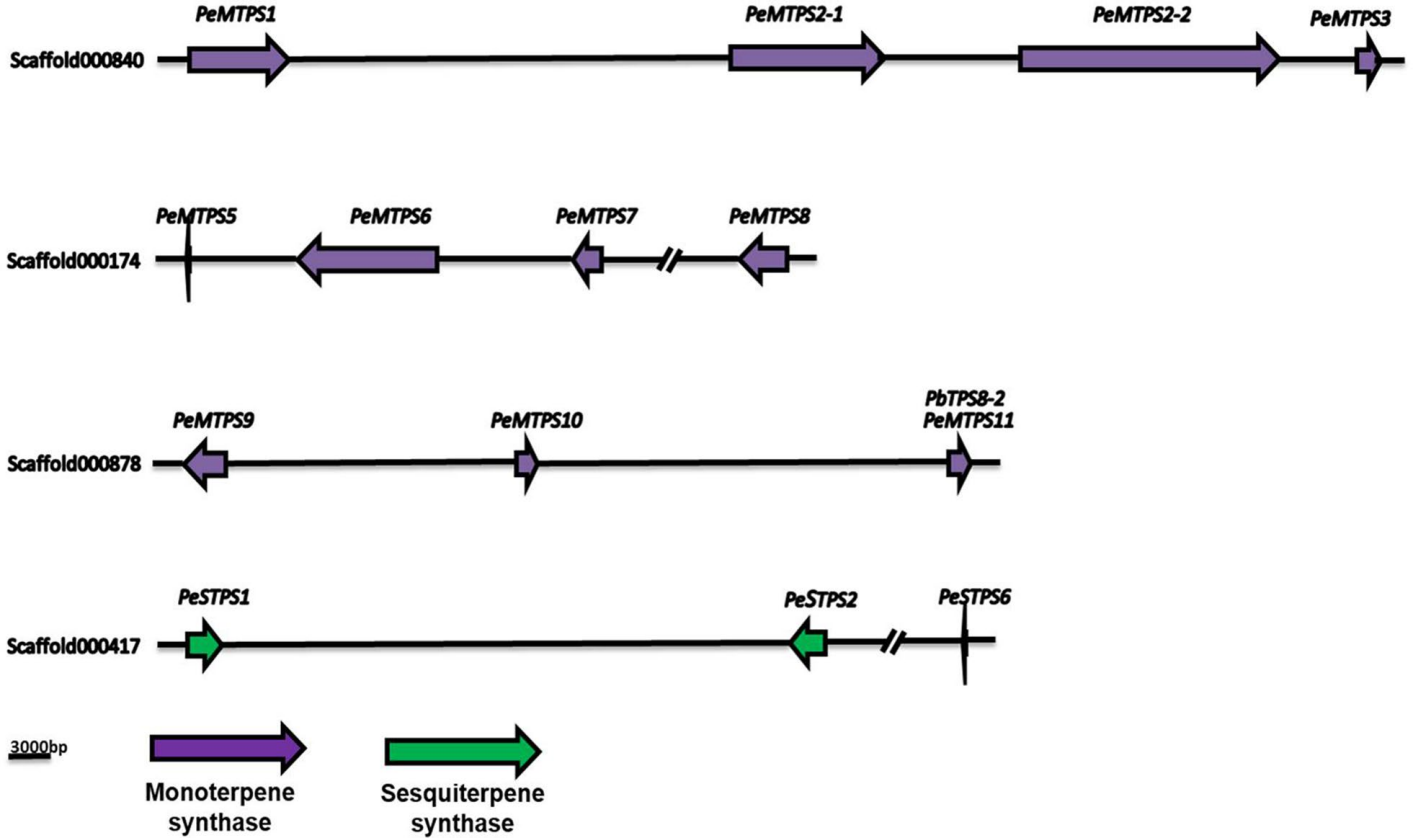
TPS of *P. equestris* form several clusters. The genes are located on the scaffolds which is form from the assembly of the *P. equestris* genome. The arrow direction shows the translation direction of genes, yellow arrow indicated the monoterpene synthase and green arrow represented sesquiterpene synthase

The large *TPS* family is divided into seven clades, from TPS-a to TPS-g, according to their protein sequence (Bohlmann et al. 1998; Dudareva et al. 2003; Martin et al. 2004). *Phalaenopsis* equestris genome has 23 TPSs belonging to TPS-a, -b, -c, e/f, -g (Figs. 3, 4). We investigated the TPS evolutionary relationship among orchids, to see whether duplication and then sub- or neo-functionalization of the *TPSs* have occurred during evolution. Cao et al. (2010) has proposed that diterpene synthases are the origin of mono- and sesqui-terpene synthases. Interestingly, the evolution of *TPSs* with other related genes may also create unexpectable mechanism. For instance, the evolution pathways of *TPS/CYP* pairs are different in monocot and dicot: *TPS*/*CYP* pairs duplicated with ancestral *TPS*/*CYP* pairs as template to evolve in dicot, but evolutionary mechanism of monocot showed genome rearrangement of *TPS* and *CYP* individually (Boutanaev et al. 2015). Moreover, *TPS* cluster density showed that *TPSs* occupied 0.008 gene/Mb in *P. patens* genome, 0.07 gene/Mb in Sorghum, and 0.3 gene/Mb in Arabidopsis and *Vitis vinifera* (Chen et al. 2011). *Phalaenopsis* tends to have dense TPS cluster compared to others, with 1.4 genes/Mb found in *P. equestris* and 2.09 genes/Mb in *Dendrobium*. It is possible that the higher density of TPSs genes in both *Phalaenopsis* and *Dendrobium* are related to that they both are CAM plant, and requires the TPS cluster to synthesize terpenoid compounds quickly for them to circumvent the adverse environment.

The *TPS* number, expression pattern, gene structure and phylogeny relationship among orchids may reveal the initial architecture of the *TPS* genes role in the orchids. Based on functions of these TPSs, mono-, di- and sesquiterpene synthases are found in orchids, Arabidopsis, and *Oryza sativa*. Total numbers of TPSs found in *P. equestris* are lesser than in *Arabidopsis*, rice, and *Dendrobium*, but the annotated monoterpene synthases are more than the others. *Dendrobium* seems to have significant larger group of annotated diterpene synthases than *Arabidopsis*, rice, and even the closest related *P. equestris* (Table 3).

**Table 3:** The annotated function of *TPS* of *Arabidopsis*, *Oryza* and orchids

Annotated function	*Arabidopsis thaliana*	*Oryza sativa*	*Dendrobium catenatum*	*Phalaenopsis equestris*
Monorpene synthase	7	2	1	13
Sesquiterpene synthase	22	18	14	7
Diterpene synthase	3	12	21	3

The genes related in terpene synthesis are usually found to be lined together, forming functional clusters in plants (Matsuba et al. 2013). The functional clusters of *TPS* genes are already found in several species, such as *Eucalpyus*, *solanum*, *Vitis vinifera*, *Arabidopsis,* rice, etc. (Shimura et al. 2007). Based on functions of these TPSs, mono-, di- and sesquiterpene synthases are found in orchids, Arabidopsis, and *Oryza sativa* (Table 3). In some plant, such as *Eucalyptus*, only the *TPSs* in same subfamily would form clusters (Külheim et al. 2015). Moreover, the TPS can form cluster with genes that are related within the same biosynthesis pathway. In *solanum*, TPS form functional cluster with *cis*-prenyl transferase (Matsuba et al. 2013). In our analysis of *P. equestris* genome, total 23 TPSs were found and predicted as mono-, di-, and sesqui-terpene synthase. In addition, those TPSs form clusters on four different scaffolds, and each scaffold only has the terpene synthase with the same function (Fig. 4).

## Secondary metabolomics

Plants produce assorted specified metabolites, but the biosynthesis genes responsible for the production and regulation of these metabolites stay mostly unknown, hampering attempts to monitor plant pharmacopeia. Assumed that genes encompassing particular metabolites pathways display environmentally reliant co-regulation, we supposed that genes participated in a specified metabolites biosynthesis pathway are arranged in strong relations (modules) in gene coexpression networks, accelerating their discovery (Wisecaver et al. 2017). Many geneticists have developed an effective method for identifying the plant genes that produce the chemical compounds to protect plants from predation, and it is a natural source of many important drugs.

### Floral scent

Floral volatile compounds (VOCs) have been characterized in several orchids, such as lilac aldehydes in *Platanthera bifolia*, phenylacetaldehyde in *Gymnadenia odoratissima*, the green-leaf volatiles in *Epipactis helleborine*, chiloglottones in *Chiloglottis*, 1-octen-3-ol in *Dracula lafleurii*, and monoterpenes in *P. bellina*.

Identification of the fragrant vandaceous orchids that are endemic to Malaysia is performed by using similar strategy to excavate probable fragrance-related EST-SSRs as the molecular markers in the *Vanda* Mimi Palmer (Teh et al. 2011). The unique transcripts were obtained from the *Ophrys* species by using 454 pyrosequencing and Illumina (Solexa) technologies to identify genes responding for pollinator attraction (Sedeek et al. 2013).

### Medicinal orchids

To study the genes involved in alkaloid biosynthetic pathway and polysaccharide biosynthesis in *Dendrobium officinale*, an important traditional Chinese herb, 454 pyrosequencing and Illumina technology was respectively applied to generate plentiful ESTs (Guo et al. 2013; Zhang et al. 2016b). Among them, 69 sequences denoting 25 genes in the biosynthesis of alkaloid backbone are detected, and 170 and 37 genes encoding to glycosyltransferase and cellulose synthase, respectively, showed differential expression patterns (Guo et al. 2013; Zhang et al. 2016b). *Dendrobium catenatum* whole genome sequence predicted 28,910 protein-coding genes are comparable with those of *Phalaenopsis*. In *Dendrobium*, the expansion of several resistance-related genes indicate a potent immune system accountable for adaptation to a broad extent of ecological niches. In addition, the genes participating glucomannan synthase actions are generally linked to the biosynthesis of medicinal polysaccharides (Zhang et al. 2016b).

### Mycoheterotrophic metabolomics

Orchidaceae contain typical mycorrhizal plants. Their seeds possess no endosperm therefore they are devoided of nutrient supply. For seed germination and growth during seedling stage, the majority of orchid species are reliant on mycorrhizal fungi. Orchids are absolutely mycoheterotrophic (reliant on symbiotic fungi for the supply of carbon and nitrogen) during the achlorophyllous protocorm stage that follows seed germination in nature (Rasmussen and Rasmussen 2009; Fochi et al. 2017; Suetsugu et al. 2017).

Orchids are unique among plants in that during their life history stages, they require mycorrhizal symbioses with soil fungi from seed germination to adulthood. To identify the molecular process of orchid seed germination and the symbiotic orchid–fungus correlation, 454 and Illumina have been applied to explore transcriptomes derived from *Serapias vomeracea* (Perotto et al. 2014), *Cymbidium hybridium* (Zhao et al. 2014), *Anoectochilus roxburghii* (Liu et al. 2015), and *Gastrodia elata* (Tsai et al. 2016). The genes related to mycorrhizal symbiosis in autotrophic orchids and arbuscular mycorrhizal plants exist with common molecular mechanisms among various mycorrhizal types.

Proteome analysis of 2D-LC–MS/MS different nutrient changed stages of symbiotic germination has been performed in *Oncidium sphacelatum* (Valadares et al. 2014). A bidirectional carbon flow even in the mycoheterotrophic symbiosis in *Epipactis helleborine* has been indicated (Suetsugu et al. 2017). This is in contrast to most fully mycoheterotrophic and partially mycoheterotrophic species, which interact mainly with ectomycorrhiza-forming fungi, such as the *Sebacinales*, *Russulaceae* and *Thelephoraceae*, suggesting that their ultimate source of carbon is the photosynthate produced by nearby trees (Lee et al. 2015; Gebauer et al. 2016).

## Genome editing in orchids

In the past decade, new technologies for genetic modification have emerged and known as genome-editing technologies. These technologies depend on engineered endonucleases that show sequence-specific manner of DNA cleavage due to the presence of a sequence-specific DNA-binding domain or RNA sequence (Gaj et al. 2013; Carroll 2014). Via the binding of the specific DNA sequence, the engineered nucleases can proficiently cleave the targeted genes. The cleaved DNA with double-strand breaks (DSB) subsequently result in DNA repair and lead to gene modification at the target sites. The DNA repair mechanisms include homology-directed repair and non-homologous end joining breaks (Wyman and Kanaar 2006). Recently, the CRISPR (clustered regularly interspaced short palindromic repeats)/Cas (CRISPR-associated) system was derived from the adaptive immune system (type II) of prokaryotic organism (Jinek et al. 2012). CRISPRs were identified as an unusual sequence element containing a series of 29-nucleotide repeats separated with 32-nucleotide ‘‘spacer’’ sequences in *Escherichia coli* genome (Ishino et al. 1987; Wiedenheft et al. 2012), and function as RNA interference mechanism to bind and cleave target DNA. A short CRISPR RNA (crRNA), the type II CRISPR/Cas from *Streptococcus pyogenes*, can recognize a complementary strand in foreign DNA with sequence specificity. Furthermore, formation of a ribonucleoprotein complex with Cas9 nuclease to generate site-specific DSBs requires a transactivating crRNA (tracrRNA) (Walsh and Hochedlinger 2013). The modules of crRNA and tracrRNA are joined into a single RNA molecule, and termed as guide RNA (Mali et al. 2013). Effective cleavage includes the presence of the protospacer adjacent motif (PAM) in the complementary strand of foreign DNA succeeding the recognition sequence (Jinek et al. 2012).

### Genome editing in other crops

The crucial requirement of genome editing is the accessibility of accurate genomic data as well as gene functions which are mostly available in model plants. The deficiency for the genomic information on horticultural crops has significantly limited breeding efficiency. So far, several cashcrops for horticulture have been whole-genome sequenced (Bolger et al. 2014). These comprise grapevine, papaya, strawberry, sweet orange, etc. In addition, a substantial increased number of transcriptomes of many horticultural crops are also feasible. The genomics information include reference genome data, transcriptomic sequences and genomic resequencing data for several horticultural crops, may provide unrestricted targets for genome editing for depicting the gene functions, which sequentially can assist engaging the CRISPR/Cas technology to design better crops.

Plant genome editing alleviates governing worries associated to genetically modified (GM) plants. Up to now, plant protoplasts of *Arabidopsis thaliana*, tobacco, lettuce and rice have been transfected using CRISPR/Cas9 system and achieved targeted mutagenesis in regenerated plants at frequencies up to 46% (Woo et al. 2015). The genome targeted sites enclosed small insertions or deletions cannot be distinguished from the naturally occurring genetic variations, and that they are germline-transmissible (Woo et al. 2015).

### Genome editing in orchids

The two criteria for a crop to perform genome editing are the accessibility of genomic data and confirmation of gene functions as well as the availability for the platform of genetic transformation and regeneration process. So far, the two sequenced orchid genomes are *Phalaenopsis equestris* and *Dendrobium officinale*. The transformation of *D. officinale* is well established and has a shorter regeneration time. For *P. equestris*, the transformation system has been established, but the regeneration time is long, about 2–3 years. With these advantages, also being a popular medicinal plant for multiple pharmaceutical effectives such as immunomodulation, anti-oxidation, anti-fatigue, genome editing has been recently reported in *D. officinale* to knockout the expression of several lignocellulose biosynthesis genes, including C3H, C4H, 4CL, CCR and IRX (Kui et al. 2016). In addition, Kui et al., has adopted Agrobacterium to deliver the CRISPR/Cas9 construct and compare five promoters with Cauliflower mosaic virus 35S promoter, and identified their compatible promoter activities. Even though Kui et al. showed 100% success rate for the genome editing, they did not show the reduction of the content of lignocellulose in the knockout plants.

In *Phalaenopsis* orchids, the whole genome sequence is available, and the gene functions for floral morphogenesis, flower color, and floral scent are well studied. These have laid the groundwork for genome editing in *Phalaenopsis* orchids with the genetic transformation system. It is well expected that breeding of many more cultivars can be feasible via genome editing of these trait genes.

## Conclusions and perspectives

With the available whole genome sequences of *P. equestris*, *D. catenatum*, *D. officinale*, and *A. shenzhenica*, the genetic blueprint of orchids provides a fundamental knowledge of the genetic basis of orchids. Furthermore, the whole genome sequences of one of the most popular aromatic orchids, *Vanilla*, will be available soon. The efforts by many scientists to use a plethora of genome information and genomics tools will lead to a promising understanding of the biological, physiological, molecular and genetic mechanisms of orchids in years to come. Comparative genomics analysis revealed expanded RLCK-XV clade was detected in *Phalaenopsis* genome, and the investigation of their expression patterns and putative interactors should give new insights into their functions.

In addition, the genome sequences will also be an important resource for genetic transformation for molecular breeding, including molecular marker-assisted breeding (MAB), or genome-assisted breeding (Hruz et al. 2008), and the production of transgenic or genome-edited plants. These are necessary to aid orchid horticultural research and speed up the orchid breeding. The major goal for plant breeding is to select the best progenies with the desired traits, and GWAS can guild the genetic variations with the high-density SNP markers for mapping the regulatory locus of these traits. In *Phalaenopsis* orchids, several agricultural traits have been focused, the flower sizes, length of floral inflorescence, flower colors and pigmentation patterning, and floral fragrances. With the GWAS analysis, the high-density genetic map can be constructed, and the regulatory regions responsive for the important traits can be identified. Moreover, this will be benefit for the *Phalaenopsis* breeding for new varieties with the traits of interests.

## Abbreviations

BACs: bacteria artificial chromosomes
CAM: crassulacean acid metabolism
Cas: CRISPR-associated system
CRISPR: clustered regularly interspaced short palindromic repeats
ECD: extracellular domain
ePAV: expression presence/absence variation
ESTs: expressed sequence tags
FPCs: fingerprint contigs
GBS: genotyping-by-sequencing
GDPS: geranyl diphosphate synthase
GWAS: genome-wide association studies
HDR: homology-directed repair
HMM: hidden Markov model
LTRs: long terminal repeats
MAS: marker-assisted selection
NHEJ: non-homologous end joining breaks
NGS: next-generation sequenceing
PTI: pathogen-associated molecular patterns (PAMP)-triggered immunity
QTL: quantitative trait loci
RE: restriction enzyme
RLK: receptor-like kinase
SNP: single nucleotide polymorphism
SSRs: simple sequence repeats
TEs: transposable elements
TPSs: terpene synthases
VOCs: volatile compounds
WGDs: whole genome duplications
YACs: yeast artificial chromosomes
